# Experimental Models to Study Diabetes Mellitus and Its Complications: Limitations and New Opportunities

**DOI:** 10.3390/ijms241210309

**Published:** 2023-06-18

**Authors:** Beatriz Martín-Carro, Javier Donate-Correa, Sara Fernández-Villabrille, Julia Martín-Vírgala, Sara Panizo, Natalia Carrillo-López, Laura Martínez-Arias, Juan F. Navarro-González, Manuel Naves-Díaz, José L. Fernández-Martín, Cristina Alonso-Montes, Jorge B. Cannata-Andía

**Affiliations:** 1 Bone and Mineral Research Unit, Instituto de Investigación Sanitaria del Principado de Asturias (ISPA), Hospital Universitario Central de Asturias, 33011 Oviedo, Spain; 2 Redes de Investigación Cooperativa Orientadas a Resultados en Salud (RICORS), RICORS2040 (Kidney Disease), Instituto de Salud Carlos III, 28029 Madrid, Spain; 3 Research Unit, Hospital Universitario Nuestra Señora de Candelaria, 38010 Santa Cruz de Tenerife, Spain; 4 Nephrology Service, Hospital Universitario Nuestra Señora de Candelaria, 38010 Santa Cruz de Tenerife, Spain; 5 Department of Medicine, Universidad de Oviedo, 33006 Oviedo, Spain

**Keywords:** diabetes mellitus, experimental diabetic models, diabetic kidney disease

## Abstract

Preclinical biomedical models are a fundamental tool to improve the knowledge and management of diseases, particularly in diabetes mellitus (DM) since, currently, the pathophysiological and molecular mechanisms involved in its development are not fully clarified, and there is no treatment to cure DM. This review will focus on the features, advantages and limitations of some of the most used DM models in rats, such as the spontaneous models: Bio-Breeding Diabetes-Prone (BB-DP) and LEW.1AR1-*iddm*, as representative models of type 1 DM (DM-1); the Zucker diabetic fatty (ZDF) and Goto-kakizaki (GK) rats, as representative models of type 2 DM (DM-2); and other models induced by surgical, dietary and pharmacological—alloxan and streptozotocin—procedures. Given the variety of DM models in rats, as well as the non-uniformity in the protocols and the absence of all the manifestation of the long-term multifactorial complications of DM in humans, the researchers must choose the one that best suits the final objectives of the study. These circumstances, added to the fact that most of the experimental research in the literature is focused on the study of the early phase of DM, makes it necessary to develop long-term studies closer to DM in humans. In this review, a recently published rat DM model induced by streptozotocin injection with chronic exogenous administration of insulin to reduce hyperglycaemia has also been included in an attempt to mimic the chronic phase of DM in humans.

## 1. Concept, Classification and the Role of Experimental Models in the Study of Diabetes Mellitus

Diabetes mellitus (DM) is a major global health problem not only because of its high and growing prevalence, which has tripled in the last 20 years, but also because of the number of premature deaths it causes. In the year 2021, worldwide records indicated that approximately 537 million adults were living with DM, and more than one in ten of global deaths from all causes (12%; 6.7 million adults) were related to complications derived from the disease [1]. The prevalence of DM in 2021 was approximately 10.5%, an increase of 3,9% compared to 2010. Moreover, DM generates a great economic impact on health systems, around 11.5% of the global health spending according to the World Health Organization [1].

The DM is a multifactorial disease triggered by a combination of genetic, epigenetic and environmental factors. The increase in life expectancy and unhealthy lifestyle habits, such as a sedentary lifestyle and the consumption of foods rich in saturated fats and added sugars, are risk factors for the development of obesity and associated comorbidities, such as metabolic syndrome, also called insulin resistance syndrome. This scenario is considered a predictor of DM [1]. In fact, the coexistence of diabetes and obesity, known as “diabesity”, shows an alarming rise [2].

Although the disorders associated with DM are diverse, the common feature to all of them is hyperglycaemia due to a relative or complete insulin resistance and/or deficiency. This hormone, which is produced in the pancreatic β-cell in the islets of Langerhans, is essential in the control of the glucose homeostasis by facilitating the glucose uptake and metabolism in peripheral tissues [3].

Chronic hyperglycaemia leads to a variety of complications, such as neuropathy, retinopathy and nephropathy [3]. The latter, known as diabetic kidney disease (DKD), is one of the most frequent long-term complications associated to DM, and its prevalence has increased in recent years in parallel to the substantial rise in the obese and diabetic population. The DKD, with different degrees of renal impairment, occurs in approximately 40% of diabetic patients, and today, it is the main cause of chronic kidney disease (CKD) that needs renal replacement therapy [4,5].

The main factors involved in the progression of DKD are uncontrolled hyperglycaemia, dyslipidaemia, hemodynamic (glomerular hypertension), inflammatory and profibrotic changes [6]. DM courses silently in early stages [7,8], but in the long-term, it damages several vital organs such as kidneys, blood vessels, heart and bones [1,3,9,10]. The high prevalence of the DKD, its lethal complications together with the still uncomplete knowledge of its pathogenesis [4], makes necessary the use of preclinical models to better understand the disease.

DM can be classified into four general categories: type 1 DM (DM-1), type 2 DM (DM-2), gestational DM and a group of specific types of DM due to other causes including monogenic DM syndromes, diseases of the exocrine pancreas (such as cystic fibrosis and pancreatitis) and drug- or chemical-induced DM (such as post-transplantation DM). DM-1 and DM-2 are the most common forms of the disease. DM-1, also called insulin-dependent DM, is present in 5–10% of patients and is due to autoimmune pancreatic β-cell destruction by T-cells and macrophages, usually leading to a widespread and irreversible insulin deficiency. Although DM-1 can appear at all ages, it is most often diagnosed in children and young people. DM-2, also called insulin-independent DM, is present in 90–95% of patients. DM-2 is due to a progressive loss of β-cell insulin secretion which leads to the inadequate response of the body to the action of insulin, known as insulin resistance [3].

Preclinical biomedical models of DM are fundamental to improving the knowledge and management of the disease. For research purposes, the spontaneous, induced and transgenic models of DM, currently using rodents, are the most used. However, unfortunately there is no animal model that presents all the phenotypic and/or genotypic alterations of DM in humans. Most of the studies of DM performed in rodent models are focused on the development of strategies for the prevention and early treatment of DM. However, in the experimental field, there is a gap in the knowledge of the long-term complications of DM. One of the main reasons is the difficulties of maintaining animal experiments long enough to be comparable with what happens in advanced stages of DM in humans. Therefore, there is an urgent need to carry out long-term studies to investigate the maintained effect of hyperglycaemia and its impact on the target organs of the disease [11].

The scientific and regulatory aspects of the use of experimental animals are subject to strict ethical guidelines around the world based on the principles of the 3Rs (replacement, refinement and reduction), which aim to improve both the quality of science and animal welfare when the use of animals is unavoidable [12]. In recent years, the trend has been towards a reduction in the use of experimental animals, improving alternative models such as 3D, computational and mathematical models of diseases, and every day important progress are made in this field. In relation to DM, there are some studies that have used this approach to study gestational DM [13] or to identify molecular markers to assess the glucose response [14]. However, research with animal models remains a fundamental tool to better understand biological processes and human diseases and to develop therapies, especially for systemic diseases such as DM.

## 2. Available Models for the Study of DM-1 and DM-2

### 2.1. The Spontaneous DM Rat Models

Spontaneous autoimmune DM has been observed in several rodent strains [15]. These rodent models have been widely used for the study of the pathogenesis of the insulitis of DM-1. The major rodent model of spontaneous DM-1 is the Bio-Breeding (BB) rat, which includes both the T-lymphopenic diabetes-prone (BB-DP) stock and the non-lymphopenic diabetes-resistant stock [16]. Other examples of strains of inbred rats include the LEW.1AR1-*iddm* rats, as representative models of DM-1, and Zucker diabetic fatty (ZDF) and Goto-kakizaki (GK) rats, as representative models of DM-2.

#### 2.1.1. The Bio-Breeding Diabetic Rats

The Bio-Breeding diabetic-prone rats (BB-DP rats) were discovered in the 1970s at Bio-Breeding Laboratories in Canada. They originated from a spontaneous mutation in an outbred colony of Wistar rats affecting the major histocompatibility complex (MHC). The development frequency of DM-1 occurs in males and females in the same proportion, and between 50 and 90 days after birth, the rats show severe pancreatic insulitis, leading to a hypoinsulinemia state. The first manifestation of the disease is glycosuria at 8–16 weeks, and 90% of the rats develop overt DM-1 with hyperglycaemia, weight loss, polyuria, polydipsia and very severe ketoacidosis that requires exogenous insulin administration in order to survive [17,18]. Bio-Breeding diabetes-resistant (BB-DR) rats do not develop DM, and they are used as controls.

Even though the features of the BB-DP rats are similar to DM-1 in humans, an important limitation of this model is that DM is accompanied by a T-cell decrease, a disorder that does not occur in humans or in other animal models that makes it a questioned model. In addition, some promising antidiabetic drugs, such as anti-CD3 antidiabetic therapy, have shown the side effect of a decrease in the T-lymphocyte population, a finding that makes it unable to use this model for the study of this type of drugs [19,20,21,22,23]. Despite the mentioned limitations, the BB-DP rats have been widely used for to study the pathophysiology of DM and islet transplantation [24].

#### 2.1.2. The LEW.1AR1-*iddm* Rats

The LEW.1 AR1-*iddm* rats were originated from the congenic strain LEW.1AR1 by a spontaneous mutation which also affects a gene associated with MHC. DM-1 occurs in males and females in the same proportion, showing intense pancreatic insulitis that causes subsequent hypoinsulinemia. The LEW.1AR1-*iddm* rats develop a prediabetic state for approximately one week [25], and by week 8 of life, they present many of the signs and symptoms of DM-1 such as hypoinsulinemia, weight loss, hyperglycaemia, polydipsia, polyuria, glycosuria and ketoacidosis. They have a long life expectancy, a fact that makes them an ideal model for long-term studies [17,24,26].

#### 2.1.3. The Zucker Diabetic Fatty Rats

The Zucker fatty (ZF) rats are obese rats due to a mutation in the leptin receptor gene making them hyperphagic. They develop hyperlipidaemia and hyperinsulinemia; however, they maintain normal blood glucose levels and rarely progress to mild hyperglycaemia [27]. These alterations are also observed in the prediabetic state in humans, where obesity plays an important role as a risk factor for the development of DM-2. After selective crosses between ZF rats, Zucker diabetic fatty (ZDF) rats emerged which, unlike ZF, develop advanced insulin resistance and, progressively, hyperglycaemia that, at week 10, reaches values above 500 mg/dL.

The development of DM-2 occurs spontaneously, more frequently in male rats. Despite the genetic origin of the disease differing between these rats and humans, they develop similar complications as those observed in advanced stages of the human disease such as glomerular lesions, expansion of the mesangial matrix and tubulointerstitial fibrosis, among others. This model has been used to study the alterations associated to advanced stages of DM-2 [28,29,30].

#### 2.1.4. The Goto-kakizaki Rats

The Goto-kakizaki (GK) rats constitute a very popular DM-2 model that, unlike the previous model, does not present obesity nor hyperlipidaemia. They result from selective inbreeding between Wistar rats with impaired glucose tolerance. They develop hyperglycaemia, hypoinsulinemia and peripheral insulin resistance at 12 weeks of age. The exposure of the foetus of the pregnant rat to a hyperglycaemic environment seems to affect the normal development of β-cells. Thus, at birth, the rats have a reduced number of pancreatic islets. Additionally, in these rats, exercise can reduce the increase of glycemia. Therefore, this model shares some environmental factors of the human DM, such as the hyperglycaemia “in utero” and the effect of the physical activity, making it an attractive model for studies related to the prevention and treatment of DM-2. This model develops retinal, kidney and peripheral nerves abnormalities, which is useful for studying the complications associated with the disease. A factor that limits the choice of this model for research purposes is the low rate of effective pregnancies and the decreased number of rats obtained per litter [30,31,32].

### 2.2. The Surgical Induced DM Rat Models

This model is obtained by ligation of the pancreatic ducts or by partial or total removal of the pancreas. They are not used frequently due to the traumatic nature of the technique, though it is used in research related to pancreas transplantation [33,34,35].

### 2.3. The Diet-Induced DM Rat Models

The dietary models are useful for studying the prodromal period of the diabetic syndrome, and they are considered more a model of obesity than of DM. As it is difficult to induce DM in rats just by feeding them only with hypercaloric diets, the use of this model often requires the combination with other techniques, such as pharmacological (streptozotocin, alloxan) or partial nephrectomy, to accelerate kidney damage an reduce the time of establishment and progression of the disease [11].

Different dietary interventions, such as the consumption of the Mediterranean diet [36], caloric restriction [37,38], intermittent fasting [39] and the therapeutic potential of various dietary supplements [40], have shown antioxidant, anti-inflammatory and metabolic profile improvement effects, constituting non-pharmacological complementary therapeutic strategies for the prevention and treatment of obesity and DM. Table 1 lists some therapeutic dietary strategies carried out in diet-induced DM-2 rat models.

### 2.4. The Chemical-Induced DM Rat Models

Several chemical compounds have shown to be able to induce DM in animal models, and the two most widely used diabetogenic agents are alloxan [47] and streptozotocin (STZ) [48,49]. Both are cytotoxic glucose analogues that bind to pancreatic β-cell GLUT-2 transporters causing irreversible damage, leading to hyperglycaemia, β-cell necrosis and weight loss, without causing damage to other organs. These diabetogenic agents are very unstable, so the preparations must be prepared at the time they are injected (half-life: alloxan, 1–2 min; STZ, 1 h).

The main advantage of the chemically induced models is that they are simple and relatively cheap. In addition, following different protocols of the time of induction, route of administration and dose, it is possible to induce DM-1 or DM-2 [50]. The main disadvantages of these models are (a) that the human DM is rarely caused by a toxic substance; (b) the possibility that these compounds can cause toxicity in the liver and tubular cells where GLUT-2 is expressed; and (c) that a single dose can cause mortality due to ketosis associated with acute damage [51,52].

#### 2.4.1. The Alloxan Model

Alloxan is a uric acid derivative that can selectively inhibit glucose-induced pancreatic insulin secretion by inhibiting glucokinase inducing insulin-dependent DM by promoting the formation of reactive oxygen species that cause selective β-cell necrosis. The diabetogenic dose range is very narrow, and even a mild overdose can cause systemic toxicity, especially to the kidney, although the damage is reversible in the surviving animals. It can be administered intraperitoneally (i.p.), intravenously (i.v.) and subcutaneously (s.c.), and the most frequent dose in rats is 45–65 mg/Kg i.v. [47].

#### 2.4.2. The Streptozotocin Model

Streptozotocin (STZ) or [2-deoxy-2-(3-(methyl-3-nitrosoureido)-D-glucopyranose] is a nitrosourea analogue attached to a glucosamine moiety, isolated from *Streptomyces achromogenes*. The mechanism of action also involves the binding of the GLUT-2 transporter before entering in the β-cells and the nucleus where it causes alkylation and, consequently, fragmentation of the DNA promoting pancreatic β-cell necrosis, resulting in an insulin-dependent state due to insulin deprivation. Its sensitivity depends on the species, strain, sex (males are more susceptible), age and nutritional status of the animal.

The STZ can be administered i.p., i.v. and s.c., either as a single dose, between 35 and 65 mg/Kg (the most frequently used), or multiple doses, between 20 and 40 mg/Kg, during several days [49,53]. Adult rats are usually used to establish DM-1 by multiple doses (20–40 mg/Kg) during several days or a single dose of 40–200 mg/Kg. To establish DM-2, neonatal rats with a single dose (35–65 mg/Kg i.p.) or adult rats are used in which nicotinamide or fructose are added as antioxidants to protect the animals from the cytotoxic action of STZ obtaining a partial destruction of the pancreas. The use of STZ is preferred than alloxan in rats.

With the use of STZ or alloxan, the metabolic result is a DM-2 hyperglycaemia state which does not include other epigenetic/environmental factors, such as obesity, which play an important role in DM-2 in humans. To bring these models closer to the DM-2 in humans, the combination of these toxins with diets rich in fat/sugars can be used to better resemble the state of poor nutrition/overweight/obesity currently found in the DM-2 in humans [54,55].

## 3. Are the Current Rat Models to Study the Human Diabetic Kidney Disease Enough?

The diabetic kidney disease (DKD) is a very important complication of the DM. In the human DKD, clinical and/or biochemical data, shown in Table 2, are useful to determine the evolutionary course of the disease, avoiding invasive techniques [56,57].

In animal models of DM, the beginning and progress of the signs and symptoms of the Table 2 are variable. Due to the multifactorial aetiology and complex pathogenesis of the human DM, there is no animal model that mimics all the structural and functional changes observed in humans [58,59]. These limitations led the Animal Models of Diabetic Complications Consortium (AMDCC) to propose three criteria to be met by a murine model to be considered as an acceptable DKD, listed in Table 3 [60].

The three criteria are not usually met in the same animal model; however, many of them are close to the changes observed in the human DKD. As it is unlikely that a single animal model will develop all the multifactorial complications of the DM in humans, it is advisable to use the different experimental models according to the main objectives of the planned study.

Thus, the first step is to choose the most suitable animal model to answer the research questions of the study [24].

## 4. Use of Rat Models for the Study of Antidiabetic Drugs

The very promising antidiabetic therapy with anti-CD3 antibodies induces a decrease in the lymphocyte population, a fact which makes it unable to use BB-DP rats due to their lymphopenia derived from the loss of GTPase function. This limitation is not present in the LEW.1AR1-*iddm* rat model which better resembles the pathophysiological characteristics of DM-1 in humans. In addition, LEW.1AR1-*iddm* rats develop DM rapidly, without a prolonged prodromal phase, a circumstance that, added to a longer life expectancy, makes it possible to use this strain to analyse the effect of therapies before developing age-related changes.

The new DM therapeutic approaches go far beyond only lowering blood glucose levels; they aim to act on key metabolic steps, such as sodium-glucose cotransporter-2 inhibitors (SGLT2i) and glucagon-like peptide-1 receptor agonists (GLP-1RAs) [61], which in turn shows clear benefits on the cardiorenal health of the DM [10]. Thus, to study the efficacy of SGLT2i and GLP-1RA, as well as their mechanisms of action, experimental models that develop cardiorenal complications because of the diabetic condition are necessary. Below is Table 4, which compiles different models of DM in rats used in studies with SGLT2i and/or GLP-1RA.

Most of the studies included in Table 4 concentrated on the general beneficial effects of the drugs used secondary to the damage associated with short-term hyperglycaemia. It is necessary to design long-term hyperglycaemia experimental models, closer to human DM, to further study their impact in the complications of DM.

## 5. Similarity of the Histological Finding in the Experimental and Human DKD

The histologic findings of the human DKD include glomerular hypertrophy, glomerular basement membrane thickening with absence of immune deposits, mesangial matrix expansion, loss of podocytes, glomerular capillary walls thickening, nodular sclerosis (±Kimmelstiel Wilson nodules), arteriolar hyalinosis and tubulointerstitial fibrosis [77].

The absence of a uniform classification has led the Renal Pathology Society to develop a consensus classification of the glomerular histological lesions present in the DKD, listed in Table 5 [58].

Table 6 lists the kidney histological findings found in different DM rat models.

The non-uniformity in terms of the time of onset and severity of glomerular histological lesions found in the different studies in rats may be explained by the differences in the animal models/species of DM used and time of evolution of the DM, among others. Furthermore, compared to what happens in the DM in humans, the histological abnormalities are less than those observed in humans [59,82].

The DM model induced by STZ injection has been widely used to study the development and evolution of DKD. However, there is still no consensus regarding the age, dose of STZ used, the time to develop DKD, the parameters to consider a success the establishment of DKD and the end points of the experiments. Table 7 lists the details of the protocols used in studies with male Wistar rats and STZ.

The human DKD is a long-term complication of DM-1 and DM-2 that occurs in patients receiving insulin. However, none of the models previously described includes the use of insulin to correct or minimize the negative effects of hyperglycaemia. This circumstance has been acknowledged as a limitation to the study by several authors, in which potential biomarkers of platelet activation were analysed as instruments for the evaluation of thromboembolic risk in a model of long-term DM (28 weeks) induced by STZ (single dose of 60 mg/dL) [85].

To shed some light in this area, we recently developed a long-term rat model of DM in which we used long-term insulin administration in order to improve the control of the hyperglycaemia [86].

## 6. Experimental Model of DM and Partial Correction of the Hyperglycaemia Using Insulin

The model attempted to assess the effects of long-term hyperglycaemia in a chemically induced DM-1 model in which the blood glucose level was partially corrected during 24 weeks by the administration of exogenous insulin. Briefly, the experimental model of DM consisted in two groups of 4-month-old male Wistar rats (425 ± 43 g) and controls in which DM was obtained using STZ (55 mg/Kg). Rats were considered diabetics when weight loss, polyuria, hyperglycaemia, hyperglycosuria and elevated HbA1c levels were achieved. After the 24 weeks, the rats showed microalbuminuria (UACR > 30 mg/g) and hyperfiltration. Histologically, the kidneys showed structural changes, such as increased diameter of the proximal tubules, thickening of the glomerular basement membrane and denuded foot processes of the podocytes, with no changes in kidney fibrosis. The changes observed in this model allowed us to classify them as class I diabetic nephropathy, according to the classification of the Society of Renal Pathology [58]. In addition, the diabetic rats showed higher serum and urinary levels of advance glycation end-products (AGEs) and of their soluble receptors in urine (sRAGE) but lower soluble serum Klotho. A higher degree of fibrosis was observed in the heart. The control rats did not show any kind of changes in the kidney nor in the heart. In summary, despite a partial control of the hyperglycaemia using long-term administration of insulin, the diabetic rats showed kidney and heart important alterations.

## 7. Conclusions

Experimental models of DM are an essential biomedical research tool to better understand and improve the pathogenesis and management of the DM. However, unfortunately, so far there are no animal models that clearly resemble the disorders observed in the human DM. Furthermore, most investigations are centred in the early phase of DM. More studies are needed to mimic the long-term complications associated with the disease and the long-term effects of the treatment in the different organs damaged by DM. As a stimulus for hope, this review includes a summary of a recent long-term model of DM (24 weeks) induced by streptozotocin in which exogenous insulin was administered that can help to better understand some aspects of the pathogenesis and management of DM.

## Figures and Tables

**Table 1:** Different dietary interventions strategies in diet-induced DM-2 rat models.

Reference	Specie and Sex	Age (Weeks) or Weight (g)	DM Model (Induced or Spontaneous)	DM Establishment	Dietary Strategy	Intervention Period (Weeks)
[41]	SD ♂	6–8 (180–190 g)	HFD + STZ (30 mg/Kg)	10 weeks → Glc > 300 mg/dL	Caloric restriction (30%)	20
[42]	Wistar ♂/♀	2	HFD + Alloxan (150 mg/Kg)	48 h → Glc > 200 mg/dL	Malva neglecta Wallr	2
[43]	SD ♂	48	HFD + STZ (25 mg/Kg)	3 weeks → Glc > 180 mg/dL	Magnesium supplement	4
[44]	SD ♂	200–250 g	HFD + STZ (55 mg/Kg)	4 weeks → Glc > 200 mg/dL	Alfacalcidol	4
[45]	SD ♂	8 (200–250 g)	HFD + STZ (40 mg/Kg)	2 weeks → Glc > 140 mg/dL	Chromium picolinate	10
[46]	SD ♂	200–250 g	NA (110 mg/kg) + STZ (65 mg/Kg)	1 week → Glc > 250 mg/dL	Dietary flaxseed oil rich in omega-3	5

DM: Diabetes mellitus; DM-2; type 2 DM; SD: Sprague Dawley; STZ: Streptozotocin; HFD: High fat diet; Glc: glucose; NA: nicotinamide.

**Table 2:** Evolutionary course of human DKD in different stages.

Stage		Description
1		- Glomerular hyperfiltration - Kidney hypertrophy
2		- Increased GBM thickness - Increased mesangial volume - Intermittent microalbuminuria
3	Incipient DKD	- Microalbuminuria (the best non-invasive marker to determine the risk of developing DKD): UACR > 30 mg/g; albumin excretion rate > 30 mg/24 h - Normal glomerular filtration rate - Possible increase in BP
4	Established DKD	- Proteinuria > 300 mg/24 h; UACR > 300 mg/g; Albumin excretion rate > 300 mg/24 h - Diabetic glomerulosclerosis, interstitial fibrosis, tubular atrophy and arteriolar hyalinosis - Current increase in BP (3/4 of the patients) - Progressive decrease in glomerular filtration
5	Severe kidney failure	- Further progressive decrease in glomerular filtration - High BP and diabetic retinopathy present in almost all cases - Frequent cardiovascular disease - Appearance of uremic symptoms and associated complications

DKD: Diabetic kidney disease; GBM: Glomerular basement membrane; UACR: Urine Albumin-Creatinine Ratio; BP: Blood pressure.

**Table 3:** Criteria to be met by a murine model of desirable DKD, according to the AMDCC.

Criteria	Description
1	A decline in GFR greater than 50% over the lifetime of the animal
2	At least 10-fold increase in albuminuria compared to controls of the same strain, age and gender
3	Relevant histopathological changes such as mesangial sclerosis (50% increase in mesangial volume), any degree of arteriolar hyalinosis, GBM thickening (>25% compared to baseline by electron microscopy morphometry) and tubulointerstitial fibrosis.

DKD: Diabetic kidney disease; AMDCC: Animal Models of Diabetic Complications Consortium; GFR: glomerular filtration rate; GBM: Glomerular basement membrane.

**Table 4:** The main characteristics of the rat DM models used in studies with SGLT2i and/or GLP-1RA.

Reference	Specie and Sex	Age (Weeks) or Weight (g)	DM Model (Induced or Spontaneous)	Type of DM	DM Establishment	Time from DM to Treatment (Weeks)	Drug Studied	Treatment Duration (Weeks)
[62]	Wistar ♂	10	STZ (65 mg/Kg)	1	Glc > 270 mg/dL	2	SGLT2i GLP-1RA	4
[63]	Wistar ♂	2	STZ (50 mg/Kg) + myocardial infarction (coronary artery ligation)	1	3 days → Glc > 300 mg/dL	3 days	SGLT2i	4 pre- + 4 post-infarctions = 8
[64]	Wistar ♂	120–150 g	HFD + STZ (35 mg/Kg)	2	Glc > 113 mg/dL	4	SGLT2i	4
[65]	GK ♂	5	Spontaneous	2	-	-	SGLT2i	24
[66]	Wistar ♂	200 ± 20 g	HFD + STZ (30 mg/Kg)	2	-	(4 HFD) + (4 STZ + HFD) = 8	SGLT2i	4
[67]	SD ♂	8	STZ (60 mg/Kg)	1	3 days	3 days	SGLT2i	3 8
[68]	Wistar ♂	6	STZ (65 mg/Kg)	1	3 days → Glc > 270 mg/dL	3 days	SGLT2i	6
[69]	SD ♂	10–12	HFD + STZ (35 mg/Kg)	2	4 weeks → Glc > 270 mg/dL	(4 HFD) + (2 days STZ + HFD)	SGLT2i GLP-1RA	4
[70]	Wistar ♂	8	STZ (50 mg/Kg)	1	2 days → Glc > 250 mg/dL	8	SGLT2i	4
[71]	GK ♂	18–22	Spontaneous	2	-	-	SGLT2i	8
[72]	ZDF ♂	12	Spontaneous	2	-	-	SGLT2i	6
[73]	Wistar ♂	8	STZ (50 mg/Kg)	1	2 days → Glc > 270 mg/dL	8	SGLT2i	4
[74]	ZDF ♂	10	Spontaneous	2	-	-	SGLT2i	7
[75]	SD ♂	8	HFD + STZ (40 mg/Kg)	2	4 weeks → Glc > 300 mg/dL	(4 HFD) + (3 days STZ + HFD)	GLP-1RA	8
[76]	Wistar ♂	200–250 g	HFD + STZ (35 mg/Kg)	2	2 weeks → Glc > 300 mg/dL	(2 HFD) + (1 STZ + HFD	GLP-1RA	2

DM: Diabetes mellitus; GK: Goto-kakizaki; SD: Sprague Dawley; STZ: Streptozotocin; HFD: High fat diet; Glc: glucose; SGLT2i: sodium-glucose cotransporter-2 inhibitors; GLP-1RA: Glucagon-like peptide-1 receptor agonist. Note: Only SGLT2i or GLP-1RA are detailed, although many of the referenced studies look at more drugs (monotherapy or multitherapy).

**Table 5:** Consensus classification of glomerular histological lesions present in the DKD.

Class	Description
I	Mild or nonspecific changes by OM, and GBM thickening
IIa	Mild mesangial expansion in >25% of the observed mesangium
IIb	Severe mesangial expansion in >25% of the observed mesangium
III	Nodular sclerosis (at least one convincing lesion of Kimmelstiel-Wilson lesion)
IV	Advanced diabetic glomerulosclerosis (global glomerular sclerosis in >50% of glomeruli and class I to III lesions)

DKD: Diabetic kidney disease; OM: Optical microscopy; GBM: glomerular basement membrane.

**Table 6:** Histological findings at kidney level found in different models of DM in rats.

Reference	Specie and Sex	DM Induction	Type of DM	DM Establishment	Endpoint (Week)	Technique	Results
[78]	Wistar ♂	STZ 60 mg/Kg	1	3 days → Glc > 300 mg/dL	8, 12 y 16	H&E	↑ Glomerular size ↑ GBM thickness ↑ Mesangial cell size ↑ Inflammatory cell infiltration
[79]	Wistar ♂	STZ 50 mg/Kg or STZ 50 mg/Kg + NA 100 mg/Kg	1 y 2	3 days → Glc > 250 mg/dL	4	H&E and PAS	Vacuolar degeneration Tube collapse Infiltration of mononuclear cells ↑ GBM thickness
[80]	SD ♂	STZ 60 mg/Kg	1	3 days → Glc > 300 mg/dL	12	H&E	↑ GBM thickness Segmental differentiation Hypertrophic glomeruli
[81]	SD ♂	STZ 55 mg/Kg	1	3 days → Glc > 300 mg/dL	12	H&E, PAS and TEM	Mesangial matrix focal expansion Podocyte foot process effacement

DM: Diabetes mellitus; STZ: streptozotocin; NA: nicotinamide; Glc: glucose; H&E: haematoxylin and eosin; PAS: Periodic acid–Schiff; TEM: transmission electron microscopy; ↑: Increased; GBM: glomerular basement membrane.

**Table 7:** Examples of protocols used in the induction of DKD by STZ.

Reference	DM Establishment	Time from DM to DKD (Weeks)	DKD Establishment	Endpoint (Week)
[78]	72 h → Glc > 300 mg/dL	3	Proteinuria ≥ 30 mg/24 h	8, 12 y 16
[83]	7 days → Glc > 300 mg/dL	12	UACR (×5 vs. control) Protein/creatinine (×5 vs. control)	16
[79]	72 h → Glc > 250 mg/dL	-	Serum creatinine (×2) Creatinine clearance (1/2) ↑BUN Albuminuria (×10)	4
[84]	300–500 mg/dL	-	Proteinuria (×3) Creatinine clearance (×2) ↑Electrolytes (Na^+^, K^+^, Mg^+^ and Ca^2+^)	14

DKD: Diabetic kidney disease; STZ: streptozotocin; DM: Diabetes mellitus; Glc: Glucose; UACR: Urine Albumin-Creatinine; BUN: Blood urea nitrogen; ↑: Increased.

## Data Availability

Not applicable.
